# Supplementary material for: The interactome of intact mitochondria by cross-linking mass spectrometry provides evidence for coexisting respiratory supercomplexes
Source: Mol Cell Proteomics. 2017 Dec 8;17(2):216–32. doi: 10.1074/mcp.RA117.000470 (PMC5795388; doi:10.1074/mcp.RA117.000470)
Supplement: Supplemental Data [file supp_RA117.000470_133922_0_supp_23978_fzffwf.zip › spectra_annotation/mito_spectra_annotation/26-1-2-1-12-1.pdf]

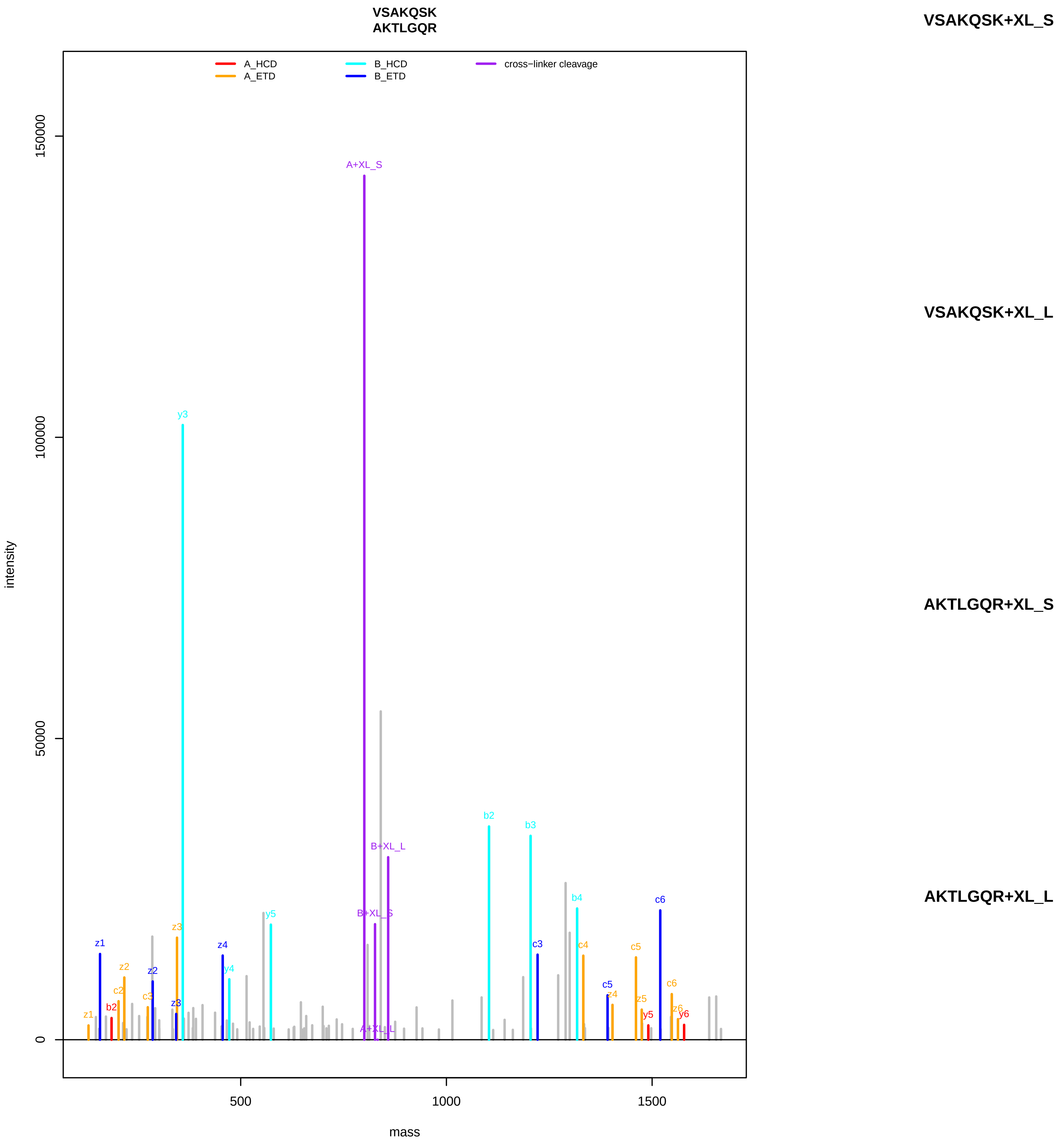

VSAKQSK
AKTLGQR
A_HCD
A_ETD
B_HCD
B_ETD
cross−linker cleavage
A+XL_S
B+XL_L
B+XL_S
A+XL_L
y3
y4
y5
b2
b3
b4
z1
z2
z3
z4
c2
c3
c4
c5
c6
z5
z6
y5
y6
c3
c5
c6
0
50000
100000
150000
500
1000
1500
intensity
mass
VSAKQSK+XL_S
VSAKQSK+XL_L
AKTLGQR+XL_S
AKTLGQR+XL_L
